# Parallel *In Vivo* DNA Assembly by Recombination: Experimental Demonstration and Theoretical Approaches

**DOI:** 10.1371/journal.pone.0056854

**Published:** 2013-02-28

**Authors:** Zhenyu Shi, Anthony G. Wedd, Sally L. Gras

**Affiliations:** 1 School of Chemistry, University of Melbourne, Parkville, Victoria, Australia; 2 Department of Chemical & Biomolecular Engineering, University of Melbourne, Parkville, Victoria, Australia; 3 Bio21 Molecular Science and Biotechnology Institute, Parkville, Victoria, Australia; Center for Genomic Regulation, Spain

## Abstract

The development of synthetic biology requires rapid batch construction of large gene networks from combinations of smaller units. Despite the availability of computational predictions for well-characterized enzymes, the optimization of most synthetic biology projects requires combinational constructions and tests. A new building-brick-style parallel DNA assembly framework for simple and flexible batch construction is presented here. It is based on robust recombination steps and allows a variety of DNA assembly techniques to be organized for complex constructions (with or without scars). The assembly of five DNA fragments into a host genome was performed as an experimental demonstration.

## Introduction

Recombinant DNA technology was the first technique proposed for targeted manipulation of DNA and has had a wide-ranging and lasting impact on many fields [1]. However, its limitations include the appearance of operational restriction sites inside the DNA sequence of interest and the challenges of *in vitro* manipulation of long DNA fragments. As a result, a defined set of rules has evolved for assembly from standardized units (bricks). The BioBrick system was one of the first attempts to standardize assembly using recombinant DNA technology [2] and has been applied in a number of synthetic biology projects [3], [4]. Improvements allowed the issues of junction scars in translation to be avoided (BglBricks; [5]) and permitted ligation of multiple DNA fragments in a single step [6].

Recently, *in vitro* recombination techniques such as sequence and ligation – independent cloning (SLIC) have been developed [7] and applied to prepare small fragments as part of the synthesis of bacterial genomes [8], [9]. It is possible to apply SLIC to the building brick strategy, but the relatively long boundaries favour pre-designed seamless assembly as SLIC is sequence independent. The efficiency of the method is similar to that of recombinant DNA technology. Hence the longer boundary is a disadvantage for small standard unit systems. An improved SLIC approach (the Gibson isothermal method) has been commercialized [10], [11]. The In-Fusion [12] and uracil-specific excision reagent (USER) [13] techniques are based on *in vitro* single-strand annealing. They employ relatively short overhangs of 15 to 30 bp, and can be modified into *in vitro* building brick systems [14].

Site-specific recombination methods have been applied widely to *in vitro* cloning. The Invitrogen Gateway system [15]–[18] uses Lambda phage attachment sequence recombination and is one of the most popular approaches as it allows multiple sites to be accessed [19]. The Cre-loxP [20]–[23] and FLP-frt [24]–[26] systems are also very efficient. However, they are less favoured for *in vitro* cloning as they do not have the binary recombination features of the attachment sequence system (such as the attachment sequence recombination: attB + attP  =  attL + attR) but remain the same after recombination. The Integron system has a similar binary mechanism to that of the Lambda phage attachment system and has been applied to the assembly of multiple genes [27].

All the above strategies are *in vitro* technologies. They rely on both high reaction and high transformation efficiencies. Standardized building brick systems aim to simplify the construction process, both for design and experiments. Since complex designs can be managed via a software platform, the reduction of experimental complexity is an important issue. One possibility is to carry out the assembly *in vivo* to avoid all *in vitro* operations. *In vivo* recombination, such as homologous recombination in *Bacillis subtillis* [28], [29] and in yeast [8], [9], [30]–[32], are more popular for the parallel assembly of large fragments. Although *in vivo* site-specific recombination is more efficient than *in vivo* homologous recombination, it has rarely been applied to DNA assembly except for some iterative systems [33]–[35] and individual integrations [36]. Lambda red-based recombinations have been applied widely in *E. coli* for gene knockout [37]–[40], for the generation of mutations [41] and for the integration of fragments into plasmids or genomes [35], [42]–[45]. The full length prophage protein recE was also reported to be able to assemble multiple fragments *in vivo* [46]. It is apparent that these extremely efficient *in vivo* recombination methods have potential in parallel DNA assembly.

A novel theoretical framework is proposed here in order to both explore the potential of parallel DNA assembly by *in vivo* recombination and to develop a standard engineering interface. It proves to be an efficient system for assembly of operons into small and medium-sized gene systems. High recombination efficiency was demonstrated by the experimental assembly of five genes.

## Materials and Methods

### *In silico* Simulation Environment

The program Vexcutor (available free from http://www.synthenome.com/) was applied to simulate and manage all *in silico* steps in the assembly process [47]. Vexcutor is a software platform that simulates molecular cloning and genetic engineering. The designs and assembly steps of all theories presented here were simulated by Vexcutor. The simulation confirms the designs are workable and provides step-by-step detailed information for the assembly procedure.

### Plasmid and DNA materials

#### Strains and Plasmids

Bacterial strains and plasmids used in the experimental demonstration are listed in Tables 1 and 2.

**Table 1:** Bacterial strains.

*Strain*	*Important Phenotypes*	*Description*	*Source*
E. *coli* Trans1T1		Initial host for assembly. Later found to be problematical due to a mutation in the HK022 attB core sequence that causes low recombination efficiency between its resulting attL and the native HK022 attR.	Transgen
E. *coli* DH5α	*recA endA*	Host for plasmid construction.	Transgen
E. *coli* BW25142	Δ*uidA4::pir-116*	Host for R6Kγ plasmid construction.	[36]

**Table 2:** Plasmids.

*Plasmid*	*Description*	*Source*
pINT-ts	pINT-ts expresses λ phage *int* when induced by heat.	[36]
pAH57	pAH57 expresses λ phage *int* and xis when induced by heat.	[36]
pAH63	pAH63 contains λ phage *attP*.	[36]
pAH69	pAH69 expresses HK022 phage *int* when induced by heat.	[36]
pAH70	pAH70 contains HK022 phage *attP*.	[36]
pAH129	pAH129 expresses Phi80 phage *int* and *xis* when induced by heat.	[36]
pAH83	pAH83 expresses HK022 phage *int* and *xis* when induced by heat.	[36]
pCMR	pCMR expresses the HK022-λ chimera *int* and xis when induced by heat. It specifically catalyzes HK022 attL and attR recombination.	This work
pCMP	pCMR expresses the HK022-λ chimera *int* and *xis*, as well as the *pir* gene.	This work
pAHP	pCMR expresses the HK022 *int,* as well as the *pir* gene.	This work
pUnitR	The assembly unit vector, HK022 attR version.	This work
pUnitP	The assembly unit vector, HK022 attP version.	This work
TARGET	The vector for converting wild-type *E. coli* to the assembly host.	This work
pUnitExR	The extraction vector, HK022 attR version.	This work
pUnitExP	The extraction vector, HK022 attP version.	This work
pUnitRHP	The assembly unit vector HK022 attR with T7 promoter and optimized mRNA structure.	This work
pUnitPHP	The assembly unit vector, HK022 attP with T7 promoter and optimized mRNA structure.	This work
pUnitRHPluxA	The library vector for luxA, HK022 attR version.	This work
pUnitRHPluxB	The library vector for luxB, HK022 attR version.	This work
pUnitRHPluxC	The library vector for luxC, HK022 attR version.	This work
pUnitRHPluxD	The library vector for luxD, HK022 attR version.	This work
pUnitRHPluxE	The library vector for luxE, HK022 attR version.	This work
pUnitPHPluxA	The library vector for luxA, HK022 attP version.	This work
pUnitPHPluxB	The library vector for luxB, HK022 attP version	This work
pUnitPHPluxC	The library vector for luxC, HK022 attP version	This work
pUnitPHPluxD	The library vector for luxD, HK022 attP version	This work
pUnitPHPluxE	The library vector for luxE, HK022 attP version	This work
pUnitExRCD	The extracted vector with assembled luxC and luxD.	This work
pUnitExPABE	The extracted vector with assembled luxA, luxB and luxE.	This work
pSB406	The template plasmid for cloning the lux genes.	[51]

#### Reagents and Bacterial Hosts

Enzymes and antibiotics used in the experimental demonstration are listed in Table S1. Primers are listed in Table S2.

#### DNA Sequences for *in silico* Simulation

All DNA sequences for simulation were obtained from GenBank and BioBricks.

**Figure 1:**
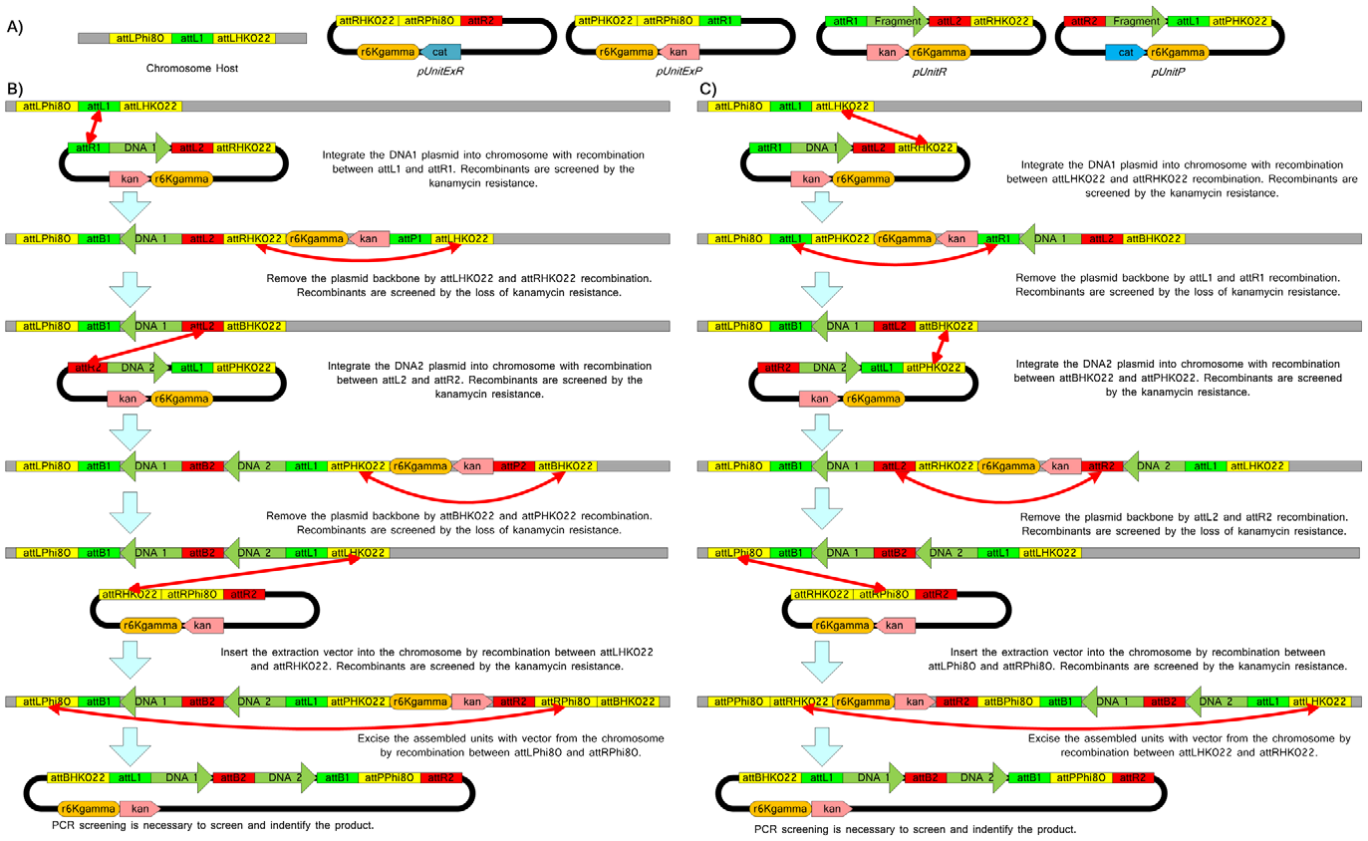
Assembly process details of SRAS. The schematic design of the Single-Selective-Marker Recombination Assembly System (SRAS) is shown here (A), consisting of two unit vectors pUnitR, pUnitP, the excision vectors pUnitExR and pUnitExP and the E.coli host chromosome. One method of I/O assembly is shown here for the assembly of two DNA fragments DNA1 and DNA 2 (B): integration occurs via reactive ends first and excision via topology breakers. A second method of I/O assembly is shown here for the assembly of two DNA fragments DNA 1 and DNA2 (C): integration occurs via topology breakers first and excision via reactive ends. The screening procedures and results are identical to B part of this figure. attLPhi80 and attPPhi80 stand for the Phi80 phage attL and attR, respectively. attLHK022, attRHK022, attBHK022 and attPHK022 stand for the HK022 phage attL, attR, attB and attP respectively. The chloroamphenicol and kanamycin resistance genes are designated cat and kan respectively. The cat gene is not employed in the schematic above but could be used as a second single selective marker in parallel rounds of DNA assembly.

A schematic diagram of the method tested experimentally using the Single-Selective-Marker Recombination Assembly System (SRAS) for parallel DNA assembly developed here is given in Figure 1. The next section describes the preparation of the experimental tools required for SRAS, followed by a description of methods involved in the experimental demonstration of SRAS.

#### Preparation of the experimental tools for experimental demonstration of SRAS

**Figure 2:**
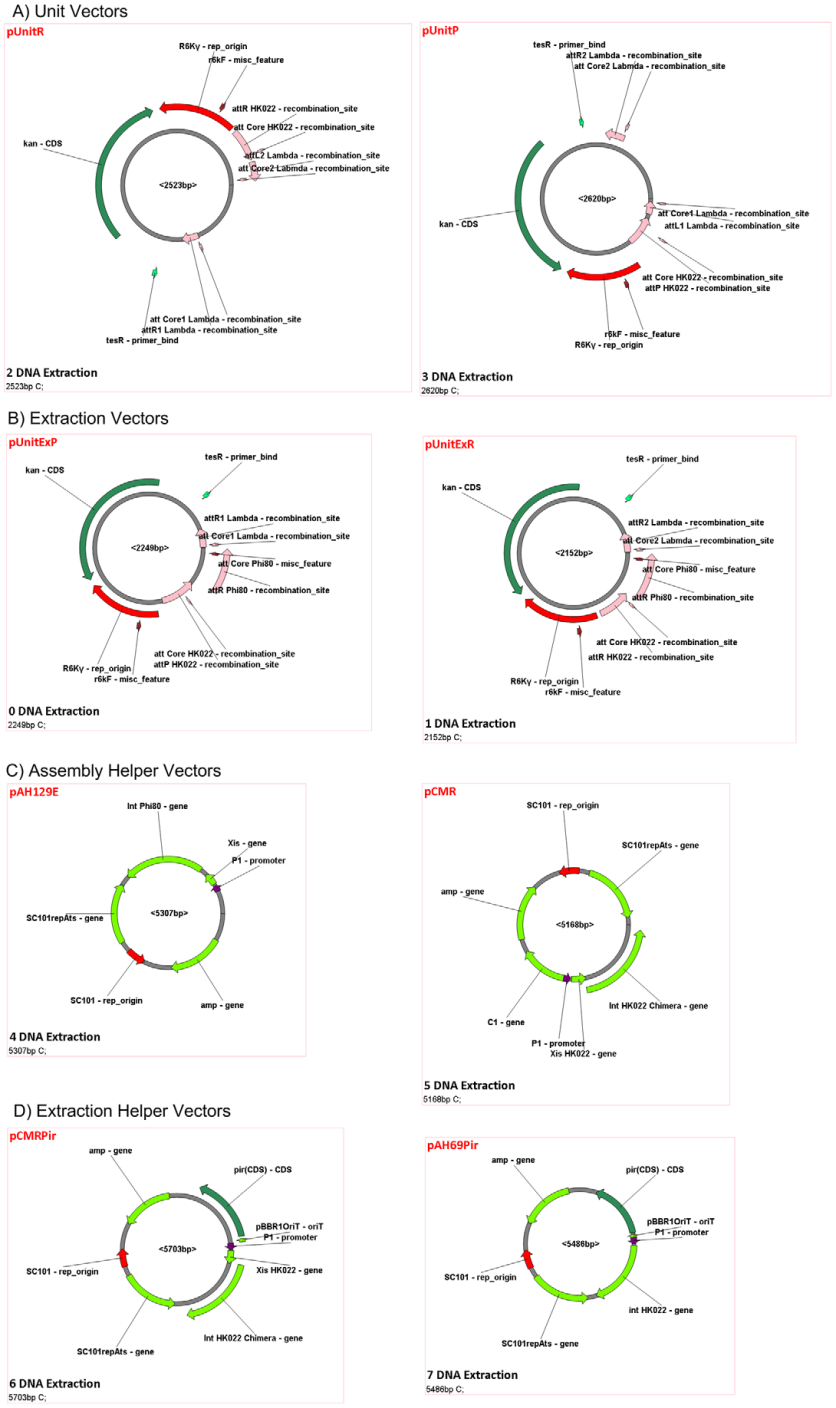
The experimental materials constructed for the demonstration of the Single-Selective-Marker Recombination Assembly System (SRAS). (A) The pUnitP and pUnitR unit vectors for constructing fragment libraries. (B) The pUnitExP and pUnitExR extraction vectors. (C) The assembly and helper Vectors that integrate the library vectors pUnitP and pUnitR into the host cell chromosome. (D) The extraction helper vectors. The helper vector contains a *pir* gene that allows the replication of the extracted vectors. (E) The host strain containing the TARGET sequence for SRAS.

A number of tools are required for the experimental demonstration of SRAS. These include the two unit plasmids (pUnitP and pUnitR), the two extraction plasmids (pUnitExP and pUnitExR), the TARGET plasmid within the host *Escherichia coli* (*E. coli*) and helper plasmids that assist with integration and excision of DNA. These tools are shown in Figure 2. The construction of these tools, integration of the TARGET plasmid and modification of unit plasmids to incorporate the T7 promoter and terminator sequences is described below.

#### Construction of unit plasmids, extraction plasmids and TARGET plasmid

**Figure 3:**
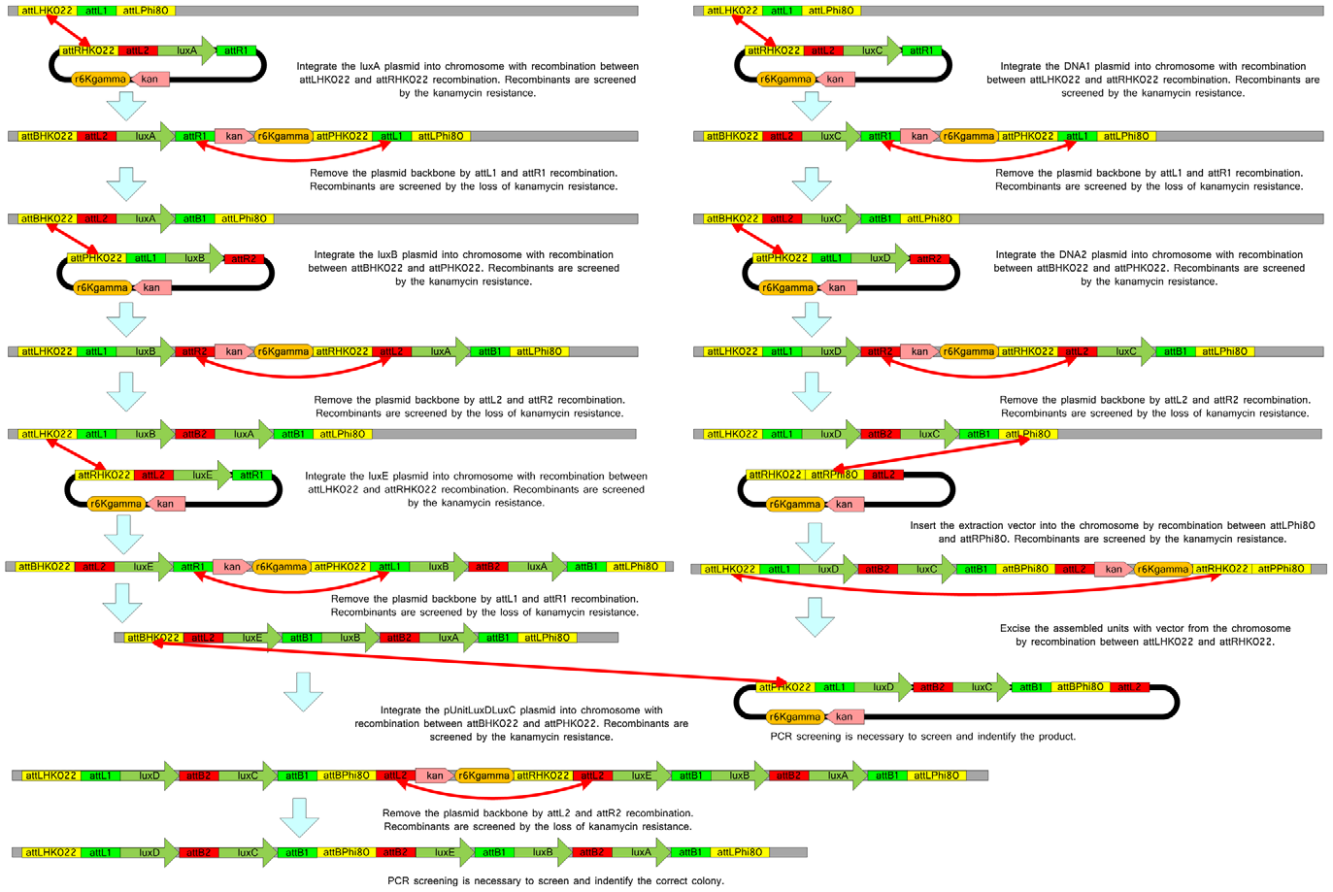
SRAS. Schematic of the Single-Selective-Marker Recombination Assembly System (SRAS) shown in Figure 1, as applied to the experimental assembly of Lux genes (A): Lux A, LuxB, LuxC, LuxD and LuxE. The screening procedures and results are identical to Figure 1. attLPhi80 and attPPhi80 stand for the Phi80 phage attL and attR, respectively. attLHK022, attRHK022, attBHK022 and attPHK022 stand for the HK022 phage attL, attR, attB and attP respectively. The chloroamphenicol and kanamycin resistance genes are designated cat and kan respectively.

A series of short DNA components including the kanamycin-resistant operon and R6Kgamma from pKD13, the chloramphenicol-resistant operon (consisting of the *cat* gene and its promoter) derived from pKD3, the synthesized lambda attL1, attL2, attR1 and attR2, the HK022 attP and attR cloned from CRIM plasmid pAH70 and its integration product, the phi80 attL and attR cloned from the integration product of the CRIM plasmid pAH153, and a cloning site region from pBHR68 [48] were assembled into the two unit plasmids (pUnitP and pUnitR), the two extraction plasmids (pUnitExP and pUnitExR) and TARGET plasmid by recombinant DNA technology (Figure 3). The detailed procedures for the construction of these plasmids and sequences can be found in the corresponding Vexcutor file (vxt file; Construction of the Unit and Extraction Plasmids.vxt), which shows the individual steps involved in the construction of each of these plasmids and the sequences *in silico* (to view download the program from http://www.synthenome.com/ as described above and open the File S1).

#### Construction of the helper plasmid

The helper plasmid, pCMR, for integration was constructed by fusing the first 330 codon of HK022 Int (pAH83) with the codons from the Lambda Int (pAH57), because this hybrid Int gene can specifically catalyze the LxR reaction of HK022 attL and attR [49], [50]. Plasmids pCMRPir and pAH69pir were constructed by inserting the pir gene into pCMR and a CRIM helper plasmid pAH69. pAH129E was constructed by destroying the c1 repressor gene in the CRIM helper plasmid, pAH129. The individual steps and sequences involved in this process are also detailed in the third file File S2.Construction of the Helper Plasmids.vxt, which can be accessed as described above.

#### Integration of the TARGET plasmid

The TARGET plasmid was integrated into the *Escherichia coli* (*E. coli*) TOP10 strain by the HK022 BxP recombination; the plasmid backbone was then replaced with lambda red recombination with pKD13 to act as a template for polymerase chain reaction (PCR); finally the pKD13 kan gene was removed by pCP20 resulting in the HOST strain with the desired chromosomal sequence shown schematically in Figure 1 A. Details of the preparation of the HOST strain and sequences are provided in the second Vexcutor file in the File S1. Construction of Host Strain.vxt.

#### Modification of the pUnit vectors

A synthesized T7 promoter and terminator region was inserted into the pUnitP and pUnitR plasmids. The details of this procedure and sequences are described in the fourth Vexcutor file in the File S4. Add T7 Promoter and Terminator to the Unit Vectors.vxt. Note that the T7 sequence is omitted from Figure 1A, as it is not a necessary part of the assembly unit.

Together, the four plasmids and host chromosome shown in Figure 1A form the basic experimental tools units required to apply the SRAS.

### Experimental demonstration of the SRAS

#### Construction of the pUnit vectors pUnitLuxA-E

To demonstrate the application of SRAS, the five genes in the lux Operon (sequence luxC, luxD, luxA, luxB, luxE) in pSB406 [51] were individually subcloned into the pUnitP and pUnitR plasmids to form the five assembly units for DNA assembly named pUnitLuxA, pUnitLuxB, pUnitLuxC, pUnitLuxD and pUnitLuxE respectively. Each gene was cloned by PCR and Kanamycin resistance used to confirm the integration of each gene within the pUnit vector. The detailed individual steps involved in this cloning and sequences used are shown in the fifth Vexcutor file in the File S5.Construction of Lux Gene Libraries.vxt.

#### Construction of the Host strains HOSTLuxDLuxC and HOSTLuxELuxBLuxA

To perform the DNA assembly from these initial five pUnit vectors, the LuxC and luxD genes were first sequentially assembled into the E. coli chromosome generating the strain HOSTLuxDLuxC, as shown to the right of Figure 3. Integration of LuxC involved recombination using the attL1 and attR1 sites, followed by the removal of the pUnit backbone by recombination between attLHK022 and attRK022. Colonies were screened for Kanamycin resistance or loss of Kanamycin resistance in these two steps, as shown in Figure 3. For example, colonies in which LuxC had integrated into the host were selected on media containing Kanamycin. In the next step, following excision of the pUnit backbone, the HOSTLuxC strains were selected by first plating bacteria on media without Kanamycin to form colonies; then 12 colonies were transferred to media with Kanamycin to select those colonies that were Kanamycin sensitive. The original live colonies on Kanamycin free media were then used for subsequent rounds of integration and assembly, which followed the same sequential steps. Lux D was integrated using recombination between attL2 and attR2 and the backbone excised using recombination between attBHK022 and attPHK022, as shown in Figure 3.

The luxA, luxB and luxE genes were next sequentially assembled into the E. coli host chromosome generating strain HOSTLuxELuxBLuxA. These steps, involved the same stages described above and shown in Figure 3. Details of the individual steps and sequences are also provided in the sixth Vexcutor file in the File S6. Recombination Process.vxt.

#### Excision of the LuxDLuxC and LuxELuxBLuxA sequences

The DNA sequences HOSTLuxDLuxC and HOSTLuxELuxBLuxA were extracted from the host chromosome using recombination between the sites attLPhi80 and attRPhi80, as shown in Figures 1 and 3. These new plasmids were named pUnitLuxDLuxC and pUnitLuxELuxBLuxA respectively. PCR was used to verify the sequence of the DNA between the reactive site ends for each of the five genes. The identity of these sequences was confirmed by DNA sequencing and compared to the sequence of the individual genes cloned in the pUnit vectors pUnitLuxA-E. The sequence of the primers and sequenced region is given in the seventh Vexcutor file Sequencing results of the extracted plasmids.vxt. To view the sequences select any ‘Compare DNA box’, select view DNA and open the DNA tab. The sequence of each gene is shown in one of the two sub tabs; the region defined by the primers is selected by clicking on the schematic on the right.

#### Construction of the HOSTLuxDLuxCLuxELuxBLuxA strain

In the final step, the luxDC genes in pUnitLuxDLuxC were integrated into the chromosome of HOSTLuxELuxBLuxA strain, using the recombination between attP HK022 and attB HK022, resulting in the strain HOSTLuxDLuxCLuxELuxBLuxA (Figure 3). The integration within the host chromosome was then confirmed by PCR. The sequence of the primers and sequenced region is given in the Vexcutor file – File S6. Recombination Process.vxt. Follow the prompts given above and open box ‘103 PCR Verification’ to view the sequences. The PCR primers amplify the DNA between attR region at the end of the LuxE gene in the HOSTLuxELuxBLuxA strain and the attL region of the LuxC gene from the integrated pUnitLuxDLuxC. The length of the PCR product was also determined by electrophoresis.

## Results

### Concepts

**Figure 4:**
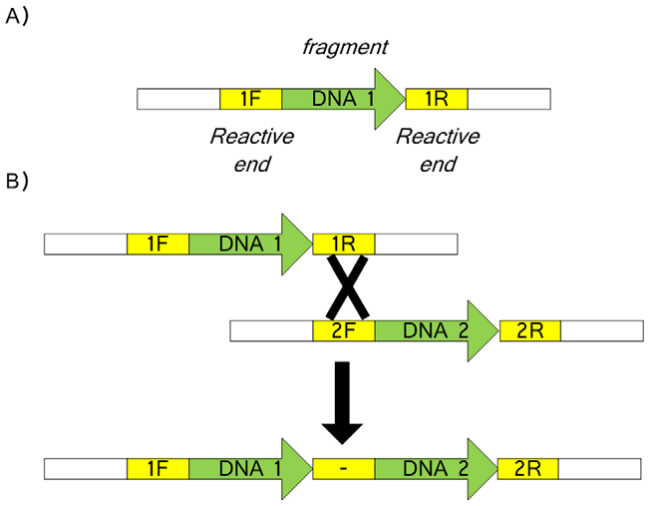
Assembly units and linear assembly. 1F, 1R, 2F and 2R are the reactive ends. (A) The conformation of a typical fragment for assembly; (B) A typical assembly reaction between two linear fragments DNA1 and DNA2. The designation ‘-‘ represents the scar left after reaction of 1R and 2F.

Effective DNA assembly requires specific and efficient reactions between any two specified ends of given *DNA fragments*. If unrelated sequences are to be excluded from assembly products, each fragment needs two tightly flanking *reactive ends* (recombination sites). A DNA fragment with two reactive ends is defined as an *assembly unit*. The assembly process for linear assembly units is simply the reaction of reactive ends. The internal sequence between the two reactive ends will not influence the assembly product. Two units in linear carriers can be assembled by one reaction between their reactive ends (see Figure 4). However, most of the stable replicable DNA molecules in the widely-used laboratory bacterial host *E. coli* are circular.

**Figure 5:**
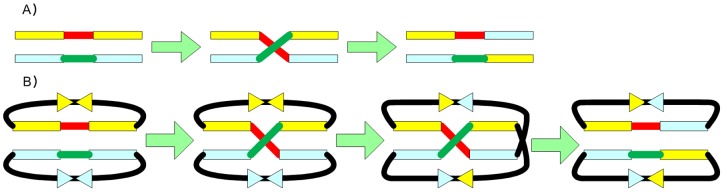
The need for topology breakers for circular plasmids. Exchange is shown occurring in the recombination of two linear fragments (shown in red and green) (A). Exchange is also shown for two circular plasmids, which requires an additional recombination (B). Topology breaker sites are shown as triangles.

An assembly unit in a circular carrier (plasmid, phagemid, chromosome) needs an additional recombination site as a *topology breaker* to ensure that combination between circular conformation plasmids is identical to that between the equivalent linear forms (Figure 5). Two linear DNA molecules exchange parts during recombination, while two circular DNA molecules insert and fuse with each other, i.e., circular DNA molecules will keep fusing if there are no topology breakers.

*Selective markers*, which are usually constitutively expressed antibiotic resistant genes, can simplify the screening process. When the assembled fragments need to be recovered from host vectors or genomes, extra *extraction sites* (which are also recombination sites) may also be required. Therefore, DNA fragments, reactive ends, topology breakers and selective markers are the necessary elements in an assembly; extraction sites are optional. In each assembly cycle, positive clones can be screened by the use of selective markers.

There are three rules for the assembly strategy proposed here:-

1. active ends must become inactive after the assembly reaction;
2. topology breakers must be reactive throughout the assembly process;
3. assembled products must have the same structure as the assembly units to allow another round of assembly to proceed.

Rules 1 and 3 are similar to the concepts developed for iGEM BioBricks [2].

#### Theory for a Single-Selective-Marker Recombination Assembly System (SRAS)

Following assembly, the selective marker needs to be removed. The iterative part of this cycle is similar to that of a previous report [34], except that the reactive ends are changed to *attL* and *attR* from the Phi80 or HK022 phage attachment sequence recombination system to satisfy Rule 1. Additional extraction vectors that enable further assembly are also required. The appropriate design of extraction vectors allows the system to obey Rule 2. Briefly, there are two ways to achieve this goal:-

1. an assembly unit can be integrated into the host vector or genome by recombination at the reactive ends; the vector backbone is then removed by a second recombination at the topology breakers;
2. an assembly unit can be integrated into the host vector or genome by recombination at the topology breakers; the vector backbone is then removed by a second recombination at the reactive ends.

The design of the assembly units developed here and schematic diagrams of the assembly steps taken to assemble DNA fragments are shown in Figure 1. There is one chromosomal host, two extraction vectors (pUnitExR and pUnitExP) and two unit vectors (pUnitR and pUnitP). Parts B and C of this figure illustrate two alternative assembly procedures for this system, where in each case two DNA fragments are assembled and then excised. Notably, the chromosomal host works as a *de facto* exclusive single-copy selective marker to ensure that all grown colonies are recombinants. As the whole assembly process features integration and excision plus extraction, it is referred to as an In/Out-Extract strategy.

### An Experimental Demonstration of In/Out-Extract SRAS

The In/Out-Extract SRAS system described in Figure 1 was selected for an experimental demonstration. Before assembly could begin, the individual pUnit vectors (pUnitP and pUnitR), the two extraction plasmids (pUnitExP and pUnitExR) and the HOST strain containing the TARGET sequence were constructed from readily available biological components. These materials, shown in Figure 1 and 2, are the minimum set of components required for the assembly of any DNA sequence from its individual genes via the In/Out-Extract SRAS system. The preparation of these materials and the subsequent assembly steps described below were tracked *in silico* using the Vexcutor program, which was designed to manage complex assembly pathways and sequences in processes such as SRAS.

The five genes in the lux Operon (luxC, luxD, luxA, luxB, luxE) were selected for the experimental demonstration of the In/Out-Extract SRAS system, as they represent a small well characterized set of prokaryotic genes that have been widely applied as a reporter system [52]. Each of the five genes were assembled into a pUnit vector, as shown in Figure 2 A, ready for the assembly process. Each gene is flanked by a T7 terminator and promoter allowing construction of the gene sequence in any order. The arrangement of a promoter with each gene potentially allows for different promoters to be integrated allowing altered levels of gene expression in later developments of this system. The sequence LuxDLuxCLuxELuxBLuxA was chosen for experimental demonstration here to differentiate from the naturally occurring order in the lux operon. As reported recently, realignment of such natural operons can result in higher performance [53].

The luxC and luxD genes were sequentially integrated into the E. coli host chromosome generating the strain HOSTLuxDLuxC, following the scheme shown in Figure 3 where the integration occurs via reactive ends (e.g. attL1 and attR1) and excision between topology breakers (e.g. attRHK022 and attLHK022). Successful colonies were selected at these steps using Kanamycin resistance or sensitivity respectively. The luxE, luxB and luxA genes were assembled sequentially in the same manner; lux A followed by lux B and then lux E, forming a second E. coli host strain HOSTLuxELuxBLuxA (see Figure 3). At this point, the two assembled sequences HOSTLuxDLuxC and HOSTLuxELuxBLuxA were extracted from the host chromosome using the recombination sites attLPhi80 and attRPhi80 generating the plasmids named pUnitLuxDLuxC and pUnitLuxELuxBLuxA respectively.

The identity of the assembled DNA sequences was confirmed by PCR using the reactive site ends flanking each lux gene (see File S7.Sequencing Results of the Extracted Plasmids.vxt for sequence details). The sequencing primers were ATF, ATR, BTF, BTR, CTF, CTR, DTF, DTR, ETF and ETR (see Table S2), corresponding to the reactive end sites attB1, attB2, and attBPhi80 flanking the lux D, C, E, B and A genes. The sequence obtained by PCR was found to match that obtained for each gene in each individual pUnit vector (Figure 2A). For example, Box 24 Compare DNA (see Vexcutor file 7 for raw sequence data) shows the sequence alignment of the LuxE gene in the assembled pUnitLuxELuxBLuxA and the original pUnitRHPLuxE starting vector. The amplified sequence is CTACATCAATAAAACTTAA for both plasmids. This PCR verification step is suggested for all assembly schemes presented here, as this step confirms DNA assembly has occurred and eliminates the possibility of incorporation of unwanted by-products that could complicate subsequent rounds of assembly.

Having confirmed the successful integration and initial assembly steps of the two and three gene sequences, the smaller of the two DNA sequences containing the luxD and luxC genes in pUnitLuxDLuxC was integrated into the chromosome of HOSTLuxELuxBLuxA strain in a single integration step (using recombination between attP HK022 and attB HK022), producing the final host strain named HOSTLuxDLuxCLuxELuxBLuxA (Figure 3). This final assembly was confirmed by PCR of the joining sequence DNA between LuxDLuxC and LuxEluxBluxA in the host genome, using primers for their reactive ends, specifically the aTTR of LuxE and the aTTL of LuxC (see File S2 for the primers and Node 103 of Vexcutor file “File S6. Recombination Process.vxt” in the Supplementary Material for raw sequence data). The length of the PCR sequence was determined to be 1.6–1.7 Kbp by gel electrophoresis, consistent with the theoretical prediction of 1682 bp.

The PCR product successfully demonstrates the assembly of the five individual gene sequences from the five original pUnit vectors. A total of six integration steps were required to assemble the five DNA sequences using a total of six pUnit vectors, with several steps occurring in parallel. This parallel approach will be a particular advantage where larger gene assemblies are required. Notably, hundreds of colonies appeared following integration at each stage of the process indicating the robust nature of this *E.coli*-based assembly process.

## Discussion

A theoretical framework is proposed for the design of an *in vivo* parallel assembly system. The components required for the In/Out-Extract SRAS approach were developed experimentally and the assembly of a set of five genes from the lux Operon (luxC, luxD, luxA, luxB, luxE) was realized. The only *in vitro* operation required, following the construction of the starting materials containing the individual genes to be assembled, is the extraction of plasmids and confirmation of sequence identity by PCR. The experimental demonstration performed here provides a proof of concept of the In/Out-Extract SRAS system using a small well-characterized operon. Theoretically, the absence of *in vitro* operations permits the assembly of large fragments via this technique with an assembly turnover cycle as short as 1–2 days for 2 fragments (2^N^ fragments in N cycles or N to 2N days, or O(log(N)) in terms of time complexity for computer science).

An inherent component of synthetic biology is the synthesis of chromosomes and genomes. Methods such as the Gibson isothermal assembly and yeast recombination have been developed for parallel DNA assembly and chromosome fabrication [8]–[11], [54]. In addition, building-brick-based methods are still popular for the construction and optimization of small and medium-sized genetic and metabolic systems [2], [4], [5], [14], [19], [27]. In metabolic engineering projects where most components need to be optimized, traditional recombinant DNA methods and building brick methods are still favoured [5], [55]–[57]. The flexibility of metabolic networks can be probed by elementary mode analysis [58] and the metabolic distribution can be analyzed by flux analysis based on chromatographic and mass spectrometric techniques [59]–[63]. However, the performance of genetic systems must be simulated currently with simplified models [64]–[67]. In addition, prediction of protein function based on known domains, patterns and motifs presently cannot predict the exact activity of an uncharacterized protein [68]–[72]. Consequently, screening of a series of enzymes that have similar activities or screening of series of different alignments and combinations of enzymes must be performed for a sound optimization study.

The advantage of the building brick method for such optimization is that the built-in adapters used for alignment and combination can be used in any order with no additional subcloning. This potentially results in lower costs for both time and labour when larger assemblies are considered. Furthermore, the native DNA fragments can be of any size, so the building brick systems have the second advantage of low sensitivity to unit size. Lastly, a lower dependency on *in vitro* operations constitutes a third advantage.

The SRAS approach demonstrated here could be further extended by the use of a Double-Selective Marker Recombination system (DRAS), a Triple-Selective-Marker Recombination Assembly System (TRAS)**,** a linear plasmid approach to double or triple selective marker systems **(**linear DRAS and linear TRAS) or a seamless linear Bi-Swap TRAS. These variations on the parallel method of DNA assembly are discussed in turn here.

**Figure 6:**
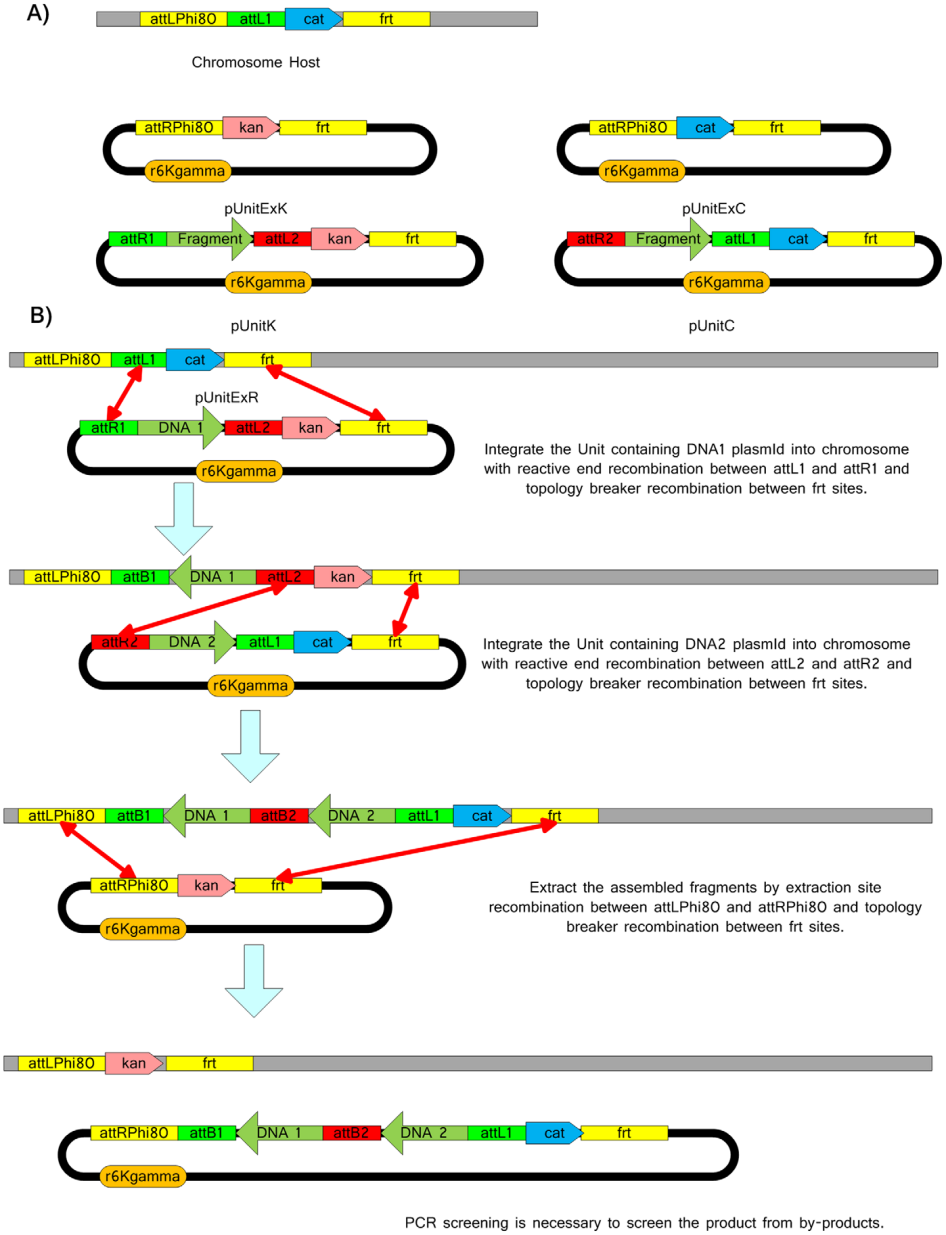
DRAS. The design of the DRAS system is shown comprising chromosome host, pUnitExK, pUnitExC, pUnitK and pUnitC (A). The Swap/Extract assembly process is shown for two fragments DNA1 and DNA2 (B). The chloramphenicol, gentamycin and kanamycin resistance genes are designated cat, gen and kan, respectively. R6Kgamma is the R6Kγ conditional replication site when PI protein is available in the cell. Frt is the frt recombination site. Mutated lambda phage attachment sequences are designated as attL1, attL2, attR1 and attR2.

When the system contains more than one selective marker, the In/Out-Extract strategy can still be implemented with minor modifications. The In/Out steps can be simplified by the swap of the two selective markers. Extraction vectors are also required. A design of this method is shown in Figure 6. It is designated as a Swap-Extract strategy. Briefly, recombination at the reactive ends and topology breaking occur concurrently and successful recombinants are screened out by substitution of the antibiotic resistant unit. The choice of topology breakers will dramatically affect the complexity of the system. Since topology breakers need to remain reactive after recombination (to obey Rule 2), frt from yeast and loxP from the P1 Phage are better options than attachment sites that feature binary recombination. In addition, the chromosomal host here has the same exclusive selective effect as occurs for the SRAS. The experimental steps involved in the DRAS approach are provided in the three Vexcutor files provided in the Files S8, S9, S10; these include the steps required to construct the DRAS host strain, a library of the lux genes previously used to demonstrate the SRAS method in a form suitable for DRAS and steps required for an equivalent demonstration of recombination.

**Figure 7:**
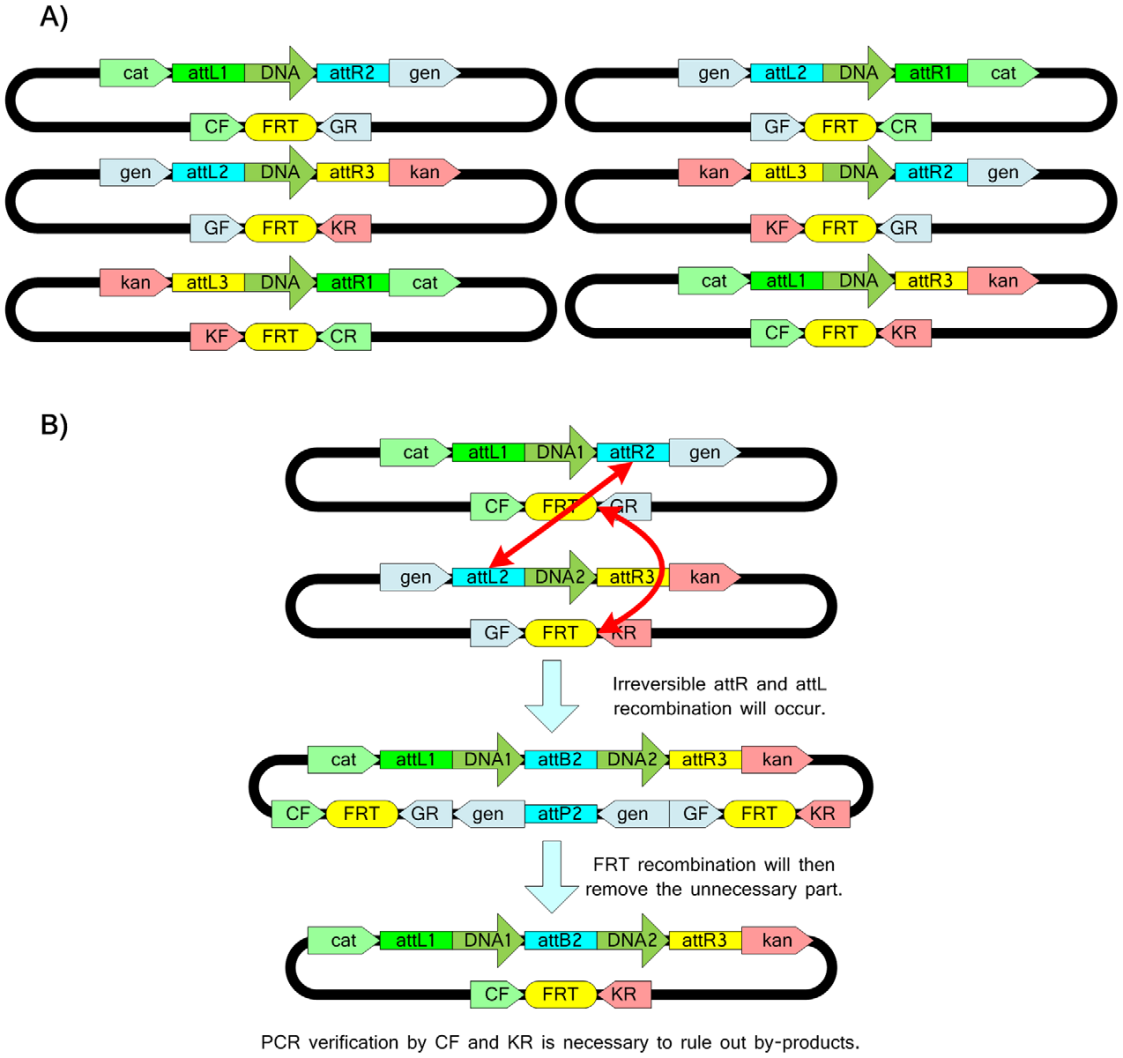
Bi-Swap TRAS. The design of Bi-Swap TRAS containing the vectors C-DNA-G, G-DNA-K, K-DNA-C, G-DNA-C, K-DNA-G and C-DNA-K (A), where CF, CR, GR, GF, KR and KF are verification primer sites. The chloramphenicol, gentamycin and kanamycin resistance genes are designated cat, gen and kan, respectively. In order to screen a recombination product with a new combination of antibiotic resistance, at least three antibiotic resistance markers must be used. Since the DNA sequence is directional and two antibiotic resistance markers must be different to perform screening, the total number of Unit plasmids is 6 (3×2). In this case, an assembly Unit vector has one Unit vector that can swap and extend its left arm or one Unit vector that can swap and extend its right arm. For example, if C-DNA1-G is the beginning unit, K-DNA2-C can be used to extend the left arm of C-DNA1-G and G-DNA2-K can be used to extend the right arm of C-DNA1-G. The assembly process of the fragments DNA1 and DNA2 (B). G-DNA2-K is used to swap and extend the right arm of C-DNA1-G. Because this is a circular plasmid system, fusion intermediates could form after recombination (but will contain all three antibiotic resistant markers). Therefore, PCR and counter-selection are considered necessary to indentify the correct products.

When three or more selective markers are available, both the In/Out-Extract and Swap/Extract strategies can be implemented. Moreover, the selective marker swap strategy can be applied between unit plasmids so that integration into the host is not necessary and the extraction step is not required. This is designated a bidirectional swap (Bi-Swap) strategy. A typical design employing the Bi-Swap strategy for the directional assembly of two DNA fragments is shown in Figure 7. The steps involved in the application of TRAS are shown in the Vexcutor file in the File S11.

**Figure 8:**
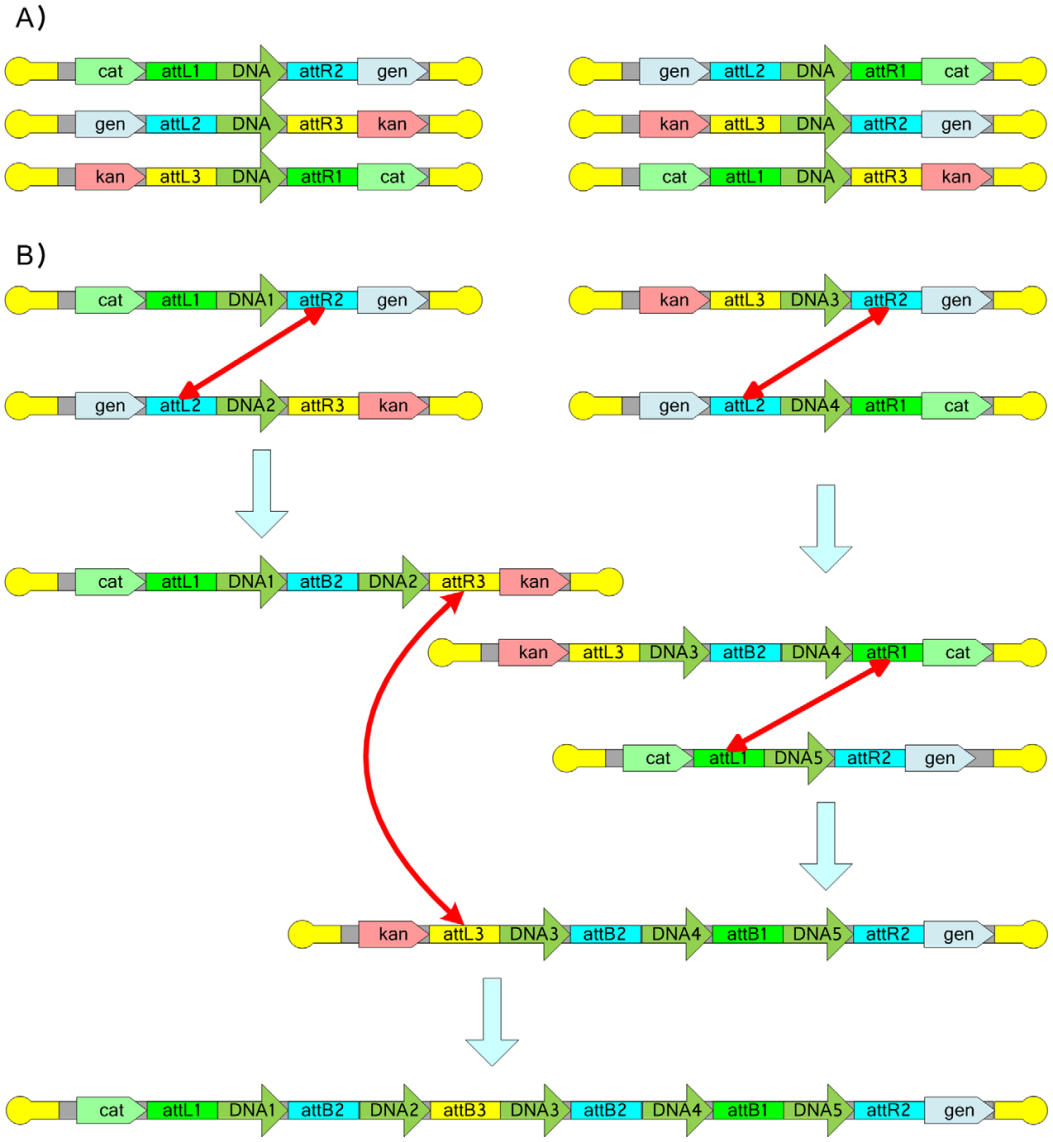
Linear Bi-Swap TRAS. The schematic design of linear Bi-Swap TRAS containing the unit vectors C-DNA-G, G-DNA-K, K-DNA-C, G-DNA-C, K-DNA-G and C-DNA-K (A). The yellow round hairpin ends represent N15 plasmids. The chloramphenicol, gentamycin and kanamycin resistance genes are designated cat, gen and kan, respectively. The assembly process for five DNA fragments DNA1, DNA2, DNA3, DNA4 and DNA5 (B). Because this is a linear plasmid system, theoretically no fusion intermediate could form after recombination. PCR and counter-selection may be necessary to indentify the correct products to avoid the false positive case where two unit plasmids exist in the same cell.

In *E. coli* and many other prokaryotes, there are also linear plasmids such as the N15 phage plasmids [73]. In the case of linear plasmids or genomes, the linear ends are ideal topology breakers because no recombination at the topology breakers is required (although telomerase can be considered as a kind of recombinase). Therefore, it is possible to use a single recombination strategy to finish the whole assembly process. Figure 8 shows a possible design for a linear Bi-Swap strategy where five DNA fragments are assembled in order. The steps involved in the linear DRAS and linear TRAS strategies are presented in a Vexcutor file in the File S12. Two more advanced Bi-Swap BioBrick examples are also presented in Figure S2 and S3. These approaches are an extension on those presented in Figure 7 and 8 that employ recombination sites as the reactive ends that drive assembly.

**Figure 9:**
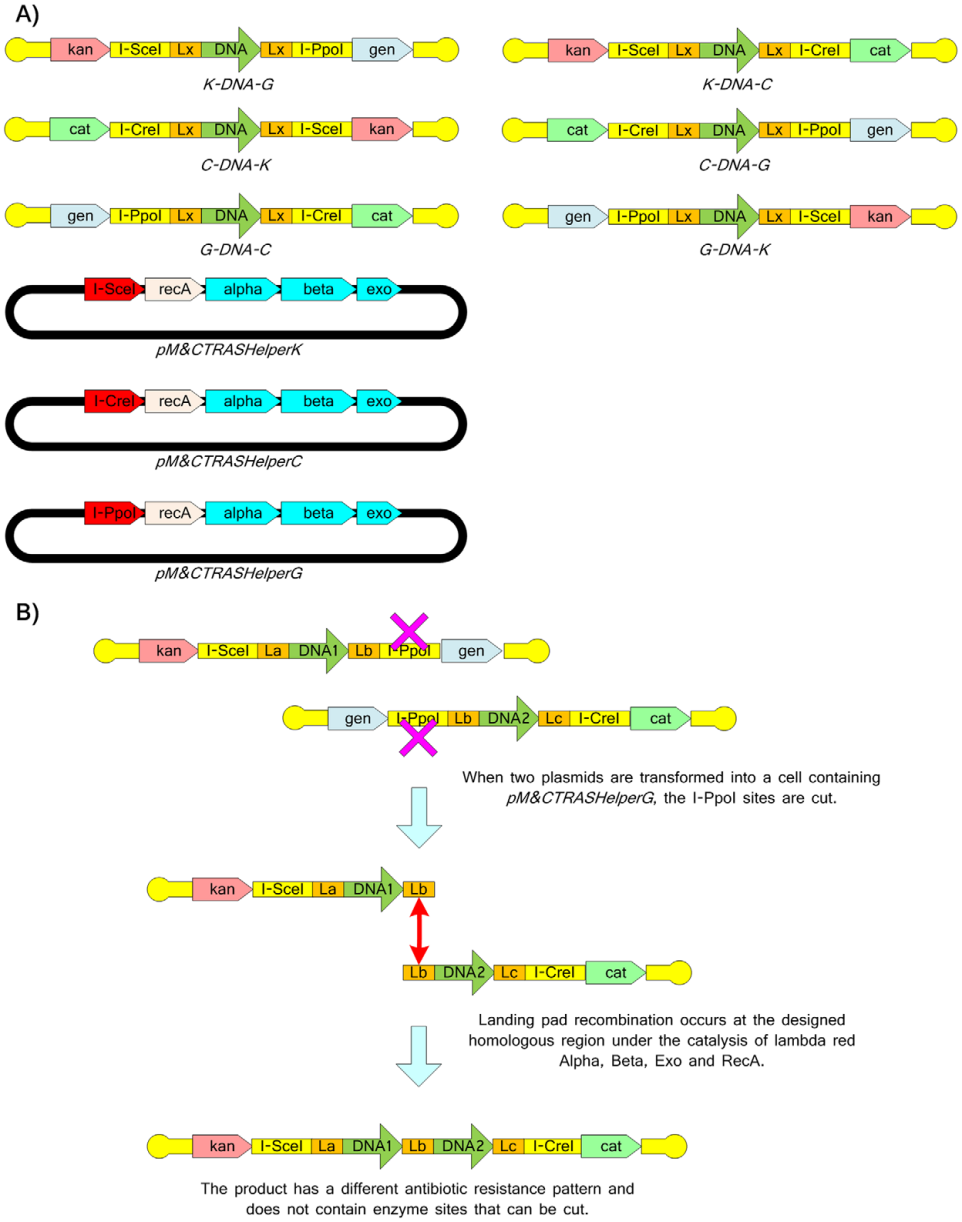
Seamless assembly system M&C (meet-and-cut) Linear-TRAS. A Design of a seamless assembly system M&C (meet-and-cut) Linear-TRAS containing the unit vectors of *C-DNA-G*, *G-DNA-K*, *K-DNA-C*, *G-DNA-C*, *K-DNA-G* and *C-DNA-K* (A), where the chloramphenicol, gentamycin and kanamycin resistance genes are designated cat, gen and kan, respectively. This system also includes the helper plasmids *pM&CTRASHelperK*, *pM&CTRASHelperC* and *pM&CTRASHelperG* for removing the kan, cat and gen markers respectively. I-CreI, I-SceI and I-PpoI in yellow rectangles indicate the homing endonuclease sites. Lx, La, Lb and Lc stand for the landing pad. I-CreI, I-SceI and I-PpoI in red triangle-ended rectangles indicate the genes encoding for the homing endonucleases. The alpha, beta and exo stand for the lambda red phage alpha, beta and exo genes. The recA stands for the E. coli recA gene. The assembly of two fragments *K-DNA1-G* and *G-DNA2-C* under the catalysis of *pM&CTRASHelperG* (B). Similar to the system described in Figure 5, PCR and counter-selection may be necessary for indentifying the correct products to avoid the false positive case where two unit plasmids exist in the same cell.

A final variation for TRAS, is a seamless model based on the MAGIC [74], Landing Pad [42] or recE [46] recombination systems. An example of a seamless linear Bi-Swap TRAS is shown for two DNA fragments in Figure 9, where the homing endonucleases expressed by the host strain are used to make site-specific double-strand breaks in the DNA. An alternative strategy is to express the required homing endonucleases on the assembly unit plasmids as each plasmid will be cut by the product of the other plasmid when both plasmids are present in the cell (Figure S1).

A further modification could be the addition of a bacterial conjugation component [75] to the TRAS vectors, allowing the assembly process to be performed by mixing two bacterial strains to facilitate conjugation. A third helper strain could be incorporated in this modification to provide this conjugation ability.

*In vivo* recombination based building brick methods presented here have the potential to be logistically simpler than other *in vitro* methods where the assembly of large numbers of fragments is required, due to the assembly of 2^N^ fragments in N cycles and shorter turnover time, as they require only plasmid transformation steps and fewer *in vitro* manipulations. The number of steps and theoretical time taken to assemble 32 fragments with different methods including the SRAS method, the Bi-Swap TRAS method (with and without conjugation), the Gibson isothermal assembly method and Recombinant DNA (or BioBrick) method is presented in Table 3. The SRAS method does not present any immediate advantages over the established methods in terms of time and number of steps, due to the use of time consuming counter selection and separate integration and excision steps. These shortcomings are overcome in both modified TRAS approaches. These differ in their need for *in vitro* steps; the Bi-Swap TRAS with conjugation is performed *in vivo*, whereas the Bi-Swap TRAS requires plasmid purification. Both TRAS approaches are quicker than the Gibson Isothermal Assembly and BioBrick methods, which rely on *in vitro* operations. The Gibson isothermal assembly method also assembles 4 fragments in each turnover cycle, while the SRAS, TRAS and Recombinant DNA method assemble 2.

**Table 3:** Number of operation steps of different strategies for assembling 32 fragments.

*Operations*	*SRAS*	*Bi-Swap TRAS with Conjugation*	*Bi-Swap TRAS*	*Gibson Isothermal Assembly*	*Recombinant DNA (BioBrick)*
Incubation (liquid)	185	63	63	41	63
Plasmid Extraction	48	0	63	41	63
Digestion	0	0	0	41	63
DNA Extraction	0	0	0	41	63
Ligation	0	0	0	0	31
Gibson Assembly	0	0	0	21	0
Conjugation	0	31	0	0	0
Electroporation	170	0	31	21	31
PCR Verification	77	31	31	21	31
Incubation (plate)	294	31	31	21	31
Number of in vivo manipulations	295	0	94	165	251
Estimated Period	24 days	5 days	5 days	8 days	10 days

Previous studies have shown that that the rate of *in vivo* recombination is near 100% for the integration of a single copy of CRIM plasmid into the *E. coli* genome [36]. This integration consists of two molecular steps: the transformation and the *in vivo* integration. In our study, we also found that the number of colonies transformed with the six Lux pUnit vectors for *in vivo* integration in the SRAS assembly was similar to that observed for the transformation of pUnit into the *E. coli* S17-1 *pir* host in which the *pUnit* plasmids can replicate, i.e., the *in vivo* integration step did not reduce cell viability, implying that the *in vivo* integration efficiency is near to 100%. In the theoretical case of Swap or Bi-Swap approaches (Figures 6 and 8), the by-products caused by the topology breaker sites (frt or loxP) can also be ruled out by counter screening for lost antibiotic resistance. Thus each of the proposed methods is expected to exhibit a high recombination efficiency – consistent with the experimental observations made here. PCR screening of the assembled genes did not reveal any artifacts from the SRAS method.

In contrast, most *in vitro* operations are less than 100% efficient as circular by-products can form, as can unpredictable mismatched products [76]. In addition, *in vitro* operations usually require demanding conditions to obtain optimum efficiency. It is apparent that the proposed *in vivo* assembly strategies have the potential of significantly increased efficiencies.

One advantage of the Bi-Swap strategy (Figures 6 and 7) is that this approach allows additional units to be added to any unit vector and vectors can be joined to any other unit. This Bi-swap model could also be useful for screening by adding antibiotic-resistant transcriptional units between the EcoRI and XbaI restriction enzyme sites and between the SpeI and PstI restriction enzyme sites. In addition, the topology breaker concepts from the new DNA assembly theory can also be applied to *in vitro* methods.

The SRAS and similar methods described here are expected to leave attachment *attB* scars with the sequence ACAAGTTTGTACAAAAAAGCAGGCT for attB1 Lambda, ACCACTTTGTACAAGAAAGCTGGGT for attB2 Lambda and AACCTTTTTCACCTAAAGTGCACC for attB HK022 between the assembled genes at a frequency of one scar per round of integration, as shown in Figures 1 and 2. These scars might prove to be an issue for a system containing many *attB* copies following the assembly of large numbers of DNA fragments because of the potential for homologous recombination. A similar issue could also occur for highly similar DNA fragments. However, due to the high recombination efficiency, the Biswap synthesis outlined here is ideal for constructing small genetic systems containing less than 20 genes. For larger systems where homologous recombination between attB scars may be a concern, the Landing pad Bi-Swap TRAS system identified in Figure S2 would be better choice and this latter system may also provide a possible strategy for the fabrication of genomic fragments.

In addition, in the case where different domains of one protein are assembled into a single reading frame or two or more genes assembled into one operon, attB scars would likely affect the open-reading frame and may affect the operon structure. The strength of the SRAS and similar methods therefore lies in making combinations and alignments of genes and the Landing Pad Bi-Swap method would be a better choice in cases where sequences have multiple domains. The approaches presented here could all prove useful, however, for difficult to combine sequences.

While an assessment of mRNA and protein expression levels of the five assembled genes from the lux Operon was beyond the scope of the present study, it is anticipated that the availability of methods for rapid DNA assembly, such as those presented here, will stimulate the study of the complementary areas of gene expression and metabolic regulation. An understanding of these fields will be essential to testing the function of the assembled DNA and integrating new pathways, such as metabolic networks or artificial chromosomes into the desired host cell.

In summary, this study has proposed and demonstrated a new framework for the design of DNA building bricks and their *in vivo* or *in vitro* assembly. The proposed system, when optimized as described in the modified TRAS and other approaches described, has the potential to reduce the labor and time associated with DNA assembly.

## Supporting Information

Figure S1 **Meet-and-cut seamless assembly system M&C Linear-TRAS.** (A) Design of a meet-and-cut seamless assembly system M&C Linear-TRAS. Linear-TRAS containing the unit vectors of C-DNA-G, G-DNA-K, K-DNA-C, G-DNA-C, K-DNA-G and C-DNA-K, where the chloramphenicol, gentamycin and kanamycin resistance genes are designated cat, gen and kan, respectively. I-CreI, I-SceI, I-NanI, I-TevI, I-PpoI and I-NitI in yellow rectangles indicate the homing endonuclease sites. I-CreI, I-SceI, I-NanI, I-TevI, I-PpoI and I-NitI in red triangle-ended rectangles indicate the homing endonuclease sites. Lx, La, Lb and Lc stand for the landing pad. (B) Assembly of two DNA fragments DNA1 and DNA2. Figure 8 has the same mechanism except that the homing endonucleases are expressed by the unit vector in this diagram rather than on a helper plasmid.
(TIF)

Figure S2 **Circular Bi-Swap BioBrick plasmid system.** (A) Design of a Circular Bi-Swap BioBrick plasmid system. The system contains the unit vectors K-DNA-G, K-DNA-C, C-DNA-K, C-DNA-G, G-DNC-G and G-DNA-K, where the chloramphenicol, gentamycin and kanamycin resistance genes are designated cat, gen and kan, respectively. XbaI, SpeI, NheI and EcoRI in rectangles indicate the endonuclease sites. Rep ori in the round-ended rectangles indicates the replication origin for each plasmid.(B) Assembly of two DNA fragments DNA1 and DNA2. Figure 6 has the same mechanism except that the reactive ends are recombination sites.
(TIF)

Figure S3 **Linear Bi-Swap BioBrick plasmid system.** (A) Design of a Linear Bi-Swap BioBrick plasmid system. The system containing the unit vectors K-DNA-G, K-DNA-C, C-DNA-K, C-DNA-G, G-DNC-G and G-DNA-K, where the chloramphenicol, gentamycin and kanamycin resistance genes are designated cat, gen and kan, respectively. XbaI, SpeI, NheI and EcoRI in rectangles indicate the endonuclease sites. Rep ori in the round-ended rectangles indicate the replication origin for each plasmid. (B) Assembly of two DNA fragments DNA1 and DNA2. Figure 7 has the same mechanism except that the reactive ends are recombination sites.
**About vxt File Format:** Please visit: http://www.synthenome.com to download the Vexcutor to view the vxt files. This program is free.
(TIF)

File S1 **Construction of the Unit and Extraction Plasmids.vxt.** Describes the construction of the Unit vectors, the extraction vectors and the TARGET vector.
(VXT)

File S2 **Construction of Host Strain.vxt.** Describes the modification of the E. coli TOP10 genome HK022 attB region with the TARGET vector fragment creating the host chromosome ready for integration.
(VXT)

File S3 **Construction of the Helper Plasmids.vxt.** Describes the construction of helper plasmids from existing CRIM vectors.
(VXT)

File S4 **Add T7 Promoter and Terminator to the Unit Vectors.vxt.** Describes the construction of the Unit vectors each with a T7 promoter and terminator.
(VXT)

File S5 **Construction of Lux Gene Libraries.vxt.** escribes the construction of the pUnit vectors, each containing one of the five genes from the lux operon.
(VXT)

File S6 **Recombination Process.vxt.** Describes the assembly process for the five lux genes cloned in pUnit vectors.
(VXT)

File S7 **Sequencing results of the extracted plasmids.vxt.** Describes the sequencing results of the extracted plasmids (i.e. pUnitLuxELuxBLuxA and pUnitLuxDLuxC). The sequence of the extracted plasmids was compared to the sequence of the same lux genes individually subcloned into the pUnit vectors. The nodes whose IDs contain “XX_B1012...”are the sequencing results of subcloned individual genes, while the nodes whose IDs contain “XX_B1102...”are the sequencing results of extracted vectors. Sequence alignments can be found in the “NA Compare”nodes.
(VXT)

File S8 **Construct DRAS Host.vxt.** Describes the construction of the DRAS host E. coli strain by modifying the Phi80 attB region.
(VXT)

File S9 **Construct DRAS lux Library.vxt.** Describes the process of subcloning the five genes of the lux operon into individual pDRASUnit vectors.
(VXT)

File S10 **DRAS Recombination.vxt.** Describes the assembly of the five lux genes subcloned in pDRASUnit vectors into the chromosome.
(VXT)

File S11 ***In Silico*** **Implementation of Bi-Swap TRAS.vxt.** Describes a possible design for a Bi-Swap TRAS strategy where five DNA fragments are assembled in order.
(VXT)

File S12 ***In Silico*** **Implementation of Linear Bi-Swap TRAS.vxt.** Describes a possible design for a linear Bi-Swap TRAS strategy where five DNA fragments are assembled in order.
(VXT)

Table S1 **Enzymes and Antibiotics.**
(DOCX)

Table S2 **Primers.**
(DOCX)
