# Supplementary figures and images for: Parallel In Vivo DNA Assembly by Recombination: Experimental Demonstration and Theoretical Approaches
Source: PLoS One. 2013 Feb 28;8(2):e56854. doi: 10.1371/journal.pone.0056854 (PMC3585241; doi:10.1371/journal.pone.0056854)

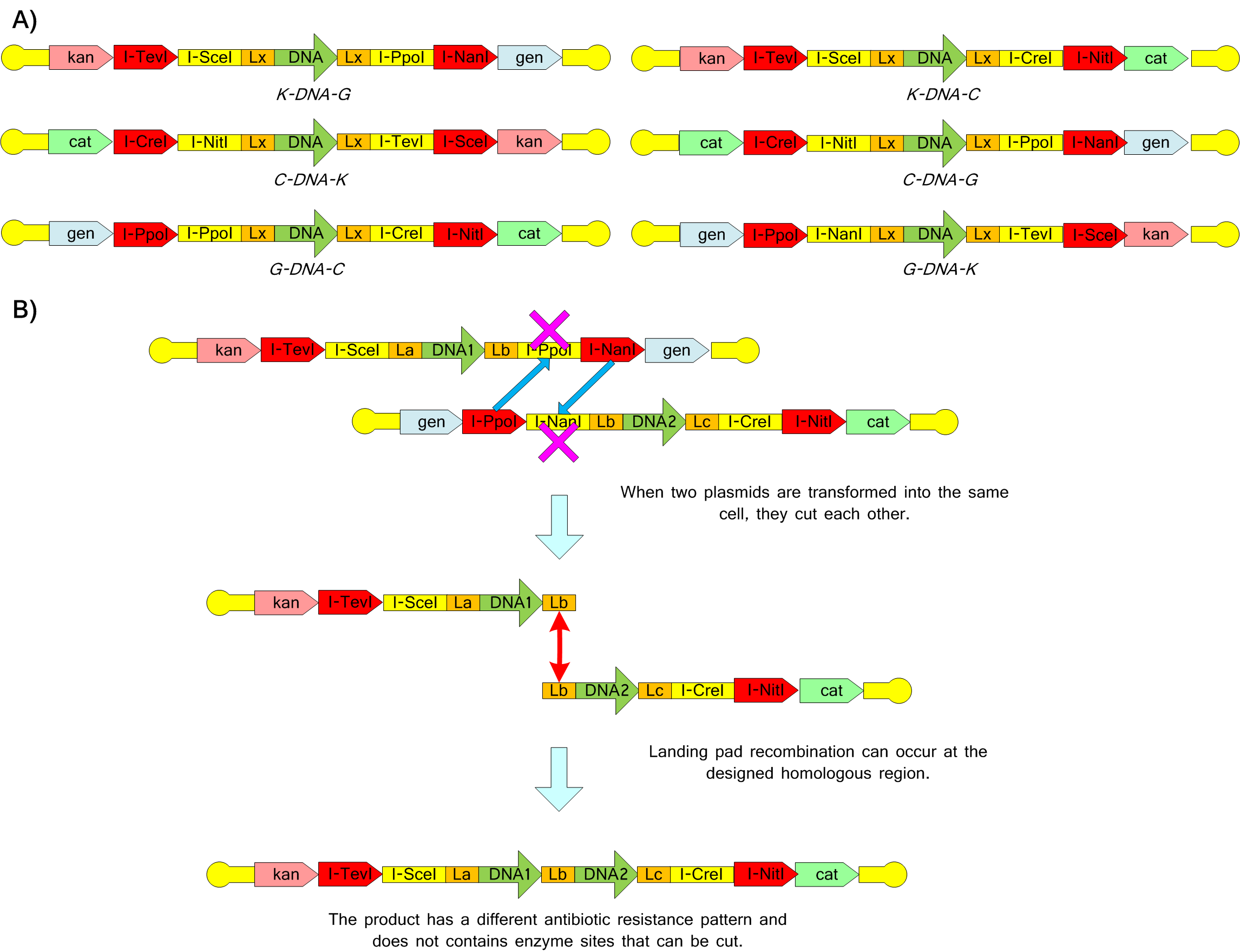

Supplement: Figure S1 — Meet-and-cut seamless assembly system M&C Linear-TRAS. (A) Design of a meet-and-cut seamless assembly system M&C Linear-TRAS. Linear-TRAS containing the unit vectors of C-DNA-G, G-DNA-K, K-DNA-C, G-DNA-C, K-DNA-G and C-DNA-K, where the chloramphenicol, gentamycin and kanamycin resistance genes are designated cat, gen and kan, respectively. I-CreI, I-SceI, I-NanI, I-TevI, I-PpoI and I-NitI in yellow rectangles indicate the homing endonuclease sites. I-CreI, I-SceI, I-NanI, I-TevI, I-PpoI and I-NitI in red triangle-ended rectangles indicate the homing endonuclease sites. Lx, La, Lb and Lc stand for the landing pad. (B) Assembly of two DNA fragments DNA1 and DNA2. Figure 8 has the same mechanism except that the homing endonucleases are expressed by the unit vector in this diagram rather than on a helper plasmid. (TIF) [file pone.0056854.s001.tif]

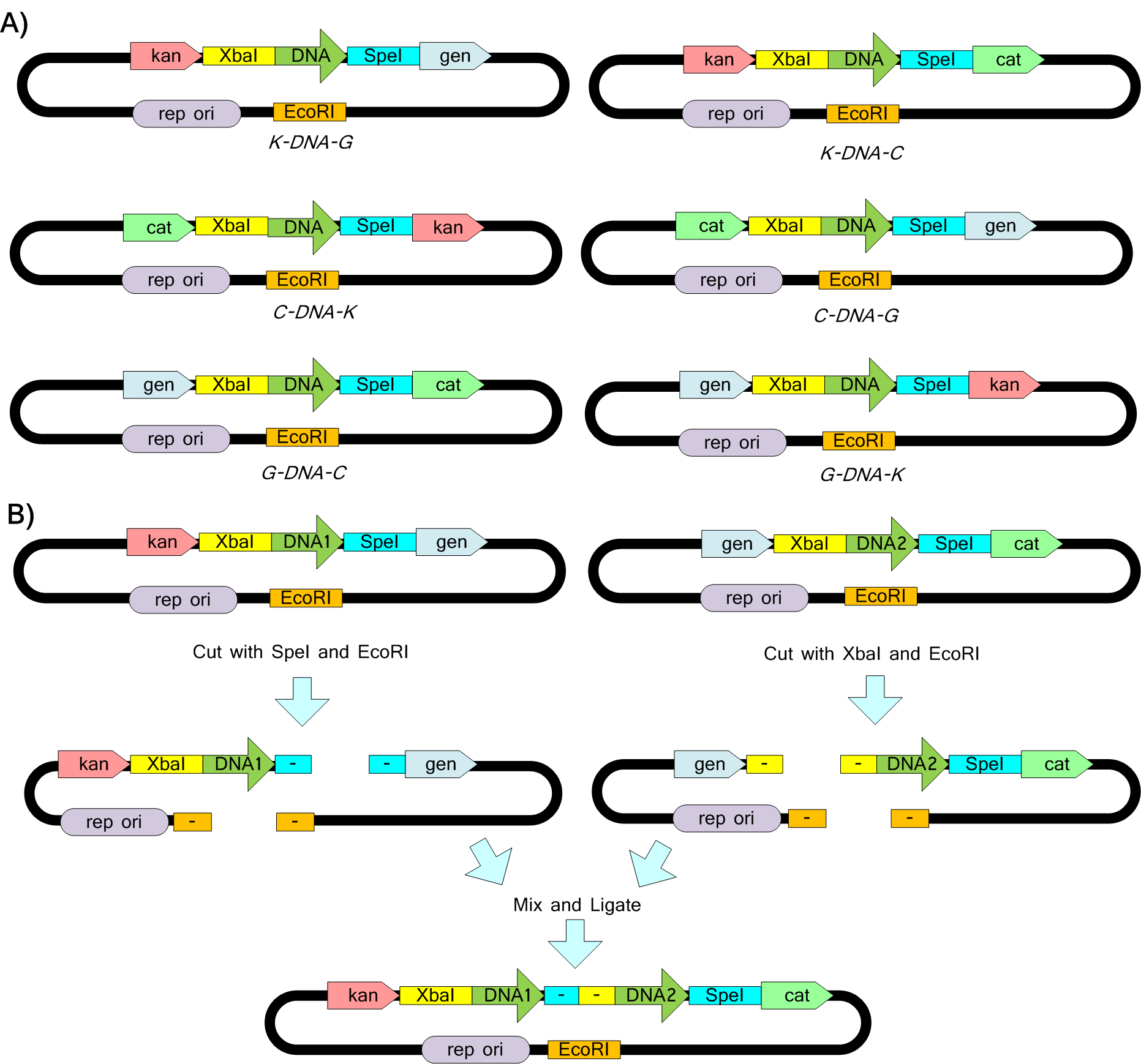

Supplement: Figure S2 — Circular Bi-Swap BioBrick plasmid system. (A) Design of a Circular Bi-Swap BioBrick plasmid system. The system contains the unit vectors K-DNA-G, K-DNA-C, C-DNA-K, C-DNA-G, G-DNC-G and G-DNA-K, where the chloramphenicol, gentamycin and kanamycin resistance genes are designated cat, gen and kan, respectively. XbaI, SpeI, NheI and EcoRI in rectangles indicate the endonuclease sites. Rep ori in the round-ended rectangles indicates the replication origin for each plasmid.(B) Assembly of two DNA fragments DNA1 and DNA2. Figure 6 has the same mechanism except that the reactive ends are recombination sites. (TIF) [file pone.0056854.s002.tif]

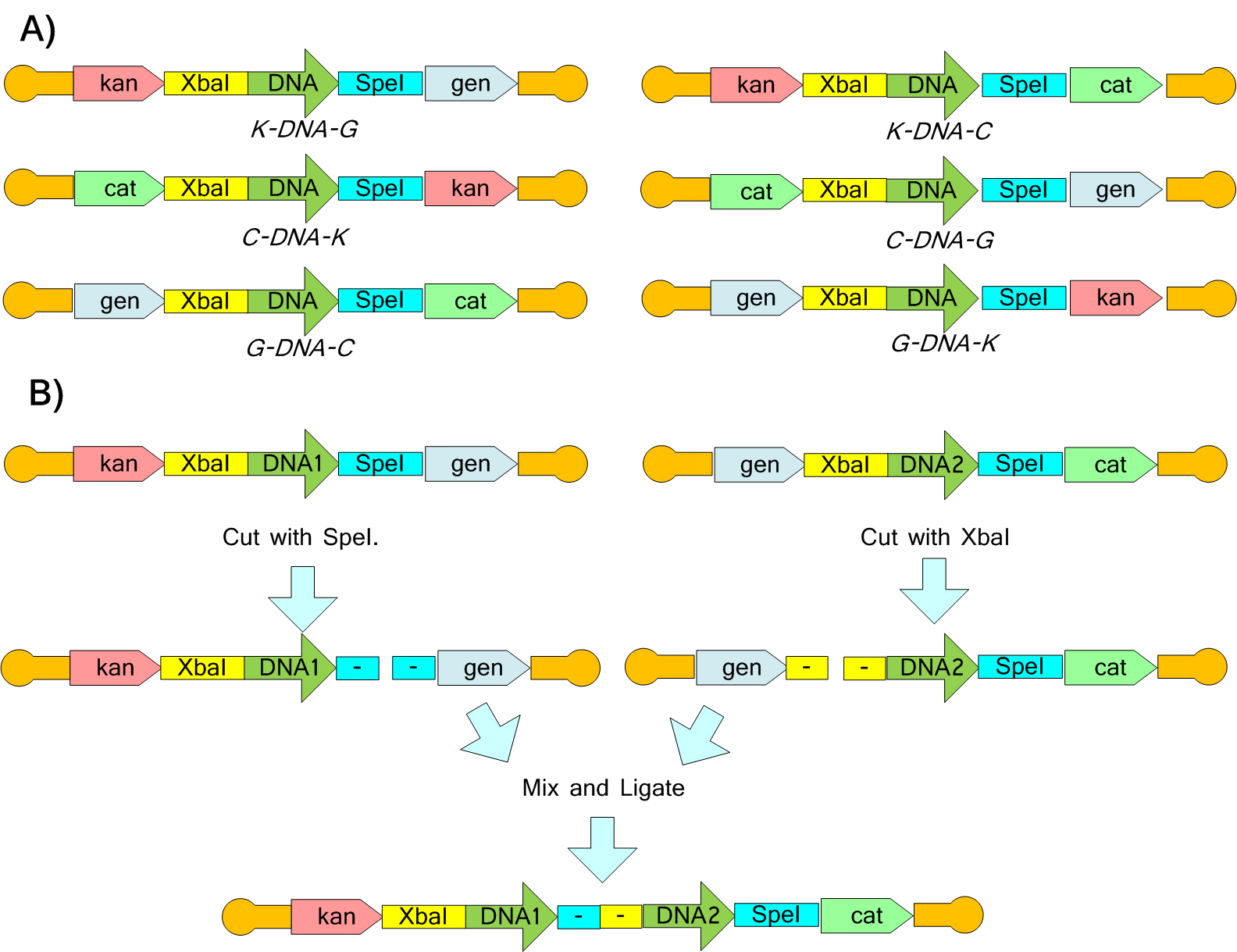

Supplement: Figure S3 — Linear Bi-Swap BioBrick plasmid system. (A) Design of a Linear Bi-Swap BioBrick plasmid system. The system containing the unit vectors K-DNA-G, K-DNA-C, C-DNA-K, C-DNA-G, G-DNC-G and G-DNA-K, where the chloramphenicol, gentamycin and kanamycin resistance genes are designated cat, gen and kan, respectively. XbaI, SpeI, NheI and EcoRI in rectangles indicate the endonuclease sites. Rep ori in the round-ended rectangles indicate the replication origin for each plasmid. (B) Assembly of two DNA fragments DNA1 and DNA2. Figure 7 has the same mechanism except that the reactive ends are recombination sites. About vxt File Format: Please visit: http://www.synthenome.com to download the Vexcutor to view the vxt files. This program is free. (TIF) [file pone.0056854.s003.tif]
