# Supplementary material for: Parallel In Vivo DNA Assembly by Recombination: Experimental Demonstration and Theoretical Approaches
Source: PLoS One. 2013 Feb 28;8(2):e56854. doi: 10.1371/journal.pone.0056854 (PMC3585241; doi:10.1371/journal.pone.0056854)
Supplement: Table S1 — Enzymes and Antibiotics. (DOCX) [file pone.0056854.s016.docx]

Supplementary Table 1. Enzymes and Antibiotics.

| *Materials* | *Source* |
| --- | --- |
| Restriction Enzymes | Fermentas |
| Easy Pfu Master Mix | Transgen |
| Easy Taq Blue Mix | Tansgen |
| Kanamycin | Sigma |
| Ampicillin | Sigma |
| Choramphenicol | Sigma |
| Gentamicin | Sigma |
