# Supplementary material for: Parallel In Vivo DNA Assembly by Recombination: Experimental Demonstration and Theoretical Approaches
Source: PLoS One. 2013 Feb 28;8(2):e56854. doi: 10.1371/journal.pone.0056854 (PMC3585241; doi:10.1371/journal.pone.0056854)
Supplement: Table S2 — Primers. (DOCX) [file pone.0056854.s017.docx]

Supplementary Table 2. Primers.

| *ID* | *Sequence* |
| --- | --- |
| hkpF | GGTACCATAAATAGCCTGAAAGGCGCTGCTAATGCTCTGTCTCAGGTC |
| hkpR | GTCGACATAATTACACTTCGTGATGGGACAAAATTGAAATCG |
| arhF | GGTACCATAAATAGCCTGAAAGGCTCAGGTCACTAATACTATCTAAGTAGTTGATTC |
| arhR | GTCGACATAATTACACTTCGTGCCGCTTTGCGACTCAACCTTT |
| all1F | GGTACCATAAATAGCCTGAAAGGCCAAATAATGATTTTATTTTGACTGATAGTGACC |
| all1R | GTCGACATAATTACACTTCGTGAGCCTGCTTTTTTGTACAAAGTTGGC |
| arl1F | GGTACCATAAATAGCCTGAAAGGCACAAGTTTGTACAAAAAAGCTGAACGAG |
| arl1R | GTCGACATAATTACACTTCGTGCATAGTGACTGGATATGTTGTGTTTTACAG |
| arl2F | GGTACCATAAATAGCCTGAAAGGCACCACTTTGTACAAGAAAGCTGAACGAG |
| all2R | GTCGACATAATTACACTTCGTGACCCAGCTTTCTTGTACAAAGT |
| aprPF | ACATTTTTGTTTTTGGGTACACAAAAGTGTA |
| aprPR | AATATTGAATCTCTATTCAGAACACTTTCTTAAATTGTCATTTG |
| aplPF | GAACACGTTTCAGTGAAACCATTTAAGAAAGTGTTCTAATTTATTAGAAATTTGC |
| aplPR | GGTGAATCACGACAAAGCGTATCAAAAAC |
| aplF | GGTACCATAAATAGCCTGAAAGGCGAACACGTTTCAGTGAAACCATTTAAGAAAG |
| aplR | GTCGACATAATTACACTTCGTGGGTGAATCACGACAAAGCGTATCAAAAAC |
| vckF | GGTACCATAAATAGCCTGAAAGGCGAGCGCTTTTGAAGCTCACGC |
| vckR | GTCGACATAATTACACTTCGTGGCGAGGCTTTGCCATGG |
| vccF | GGTACCATAAATAGCCTGAAAGGCCCTGCCACTCATCGCAGTACTGTTG |
| vccR | GTCGACATAATTACACTTCGTGATTGGTAACTGTCAGACCAAGTTTACTCATATATAC |
| clnF | ATAAGGTACCAGTCGACGCCGTCGAGGCCGATGC |
| clnR | ATATTTACACTTCGTGGGCCATGCTGGCGTCCG |
| aprF | GGTACCATAAATAGCCTGAAAGGCACATTTTTGTTTTTGGGTACACA |
| aprR | GTCGACATAATTACACTTCGTGAATATTGAATCTCTATTCAGAACACTTT |
| testF | GGCTTTACACTTTATGCTTCCG |
| testR | GGACTCCAACGTCAAAGGG |
| delF | ACGCGCATGATAGCCTCATCAATAATAAGGCTTTATGCTGTGTAGGCTGGAGCTGCTTC |
| delR | TCATCGCAGTACTGTTGTATTCATTAAGCATCTGCCGACCTGTCAAACATGAGAATTAA |
| VRTF | GAAACCAGGGCACACCAACG |
| VRTR | GTCTCAGGTCACTAATACTATCTAAGTAGT |
| VRSF | GGTGAATCACGACAAAGCGTATC |
| VRSR | AAGTTGTCGCATTATTCGCCTG |
| K1 | CAGTCATAGCCGAATAGCCTAGCCT |
| K2 | CGGTGCCCTGAATGAACTGC |
| VRTF2 | AGGTTATCAGCCGAAAATGCCG |
| VRSR2 | TGCCTGAGTGTTGTCTTTTTCCA |
| VRKF2 | GCAGTTCATTCAGGGCACCG |
| torSF | CGAACTGGGCGATGTTATT |
| luxCF | CCAGATCACCGAGTGAGGCTAATATGACTAAAAAAATTTCATTCA |
| luxCR | CCAGATCACCGTGTGTTACGGGACAAATACAAGGAACTT |
| luxDF | CCAAATCACCGAGTGAGGTAAAAAGTATGGAAAATAAATCCAA |
| luxDR | CCAGATCACCGTGTGTTAAGACAGCGAAATCGCTTG |
| luxAF | CCAGATCACCGAGTGAGGGCTCTCTATGAAATTTGGAA |
| luxAR | CCAGATCACCGTGTGCTATAATAGCGAACGTTGTTTTTCTTT |
| luxBF | CCAGATCACCGAGTGAGGAAAAAGAAATGAAATTTGGATT |
| luxBR | CCAGATCACCGTGTGCTACATGTGGTACTTTTTAATATTATCATT |
| luxEF | CCAGATCACCGAGTGAGGACAGGTATGACTTCATATGTTGATAAACA |
| luxER | CCAGATCACCGTGTGTCAACTATTAAATGCTTGGTTTAAGC |
| PXF | GACGCCGTCGGTGTAATACG |
| PXR | ACCCCTCAAGACCCGTTTAG |
| luxDF2 | CCAAATCACCGAGTGAGATAAGTTCCTTGTATTTGTCCCG |
| ATF | CATCGGTAGGCCAAATTGAG |
| ATR | CGGCGTATAGCAGAGGTGG |
| DTF | CACCCAATTTATGGCCTTCA |
| DTR | TTTTTAATCACCGCAGTCGG |
| CTF | CCAAGCGACAATAACATCCG |
| CTR | ACCCTAATCATCCGATAACGC |
| BTF | TGCATTTTTTTAATCGCCCG |
| BTR | ACATATTTCCGAGGTCCGCC |
| ETF | TGTTTACGCTGCATTTCATCC |
| ETR | CAGTAGCGGCACGAATGG |
